# Dysregulation of the complement cascade in the hSOD1^G93A^ transgenic mouse model of amyotrophic lateral sclerosis

**DOI:** 10.1186/1742-2094-10-119

**Published:** 2013-09-26

**Authors:** John D Lee, Nur A Kamaruzaman, Jenny NT Fung, Stephen M Taylor, Bradley J Turner, Julie D Atkin, Trent M Woodruff, Peter G Noakes

**Affiliations:** 1 School of Biomedical Sciences, University of Queensland, Brisbane, St Lucia QLD 4072, Australia; 2 Queensland Brain Institute, University of Queensland, Brisbane, St Lucia QLD 4072, Australia; 3 Florey Institute of Neuroscience & Mental Health, University of Melbourne, Parkville VIC 3010, Australia; 4 Department of Biochemistry, La Trobe Institute for Molecular Science, La Trobe University, Bundoora VIC 3083, Australia

**Keywords:** C1q, C4, Factor B, C3, C5, CD55, CD88, Motor neuron disease, Neuroinflammation

## Abstract

**Background:**

Components of the innate immune complement system have been implicated in the pathogenesis of amyotrophic lateral sclerosis (ALS); however, a comprehensive examination of complement expression in this disease has not been performed. This study therefore aimed to determine the expression of complement components (C1qB, C4, factor B, C3/C3b, C5 and CD88) and regulators (CD55 and CD59a) in the lumbar spinal cord of hSOD1^G93A^ mice during defined disease stages.

**Methods:**

hSOD1^G93A^ and wild-type mice were examined at four different ages of disease progression. mRNA and protein expression of complement components and regulators were examined using quantitative PCR, western blotting and ELISA. Localisation of complement components within lumbar spinal cord was investigated using immunohistochemistry. Statistical differences between hSOD1^G93A^ and wild-type mice were analysed using a two-tailed *t*-test at each stage of disease progression.

**Results:**

We found several early complement factors increased as disease progressed, whilst complement regulators decreased; suggesting overall increased complement activation through the classical or alternative pathways in hSOD1^G93A^ mice. CD88 was also increased during disease progression, with immunolocalisation demonstrating expression on motor neurons and increasing expression on microglia surrounding the regions of motor neuron death.

**Conclusions:**

These results indicate that local complement activation and increased expression of CD88 may contribute to motor neuron death and ALS pathology in the hSOD1^G93A^ mouse. Hence, reducing complement-induced inflammation could be an important therapeutic strategy to treat ALS.

## Background

Amyotrophic lateral sclerosis (ALS) is a late-onset neurodegenerative disorder characterised by selective loss of upper motor neurons within the motor cortex, and of α-motor neurons of the spinal cord and brainstem [1]. This results in symptoms of muscle weakness and atrophy of skeletal muscles, leading to paralysis and eventual death due to failure of respiratory muscles [2]. The mechanisms leading to ALS are still unclear but there are compelling data that suggest neuroinflammation may contribute to the disease progression of ALS [1,3,4]. These data include the presence of reactive microglia and astrocytes, infiltration of T lymphocytes and upregulation of cyclooxygenase 2 and prostaglandin E_2_ in the spinal cord of ALS patients and animal models [5-9]. The classical complement system is also implicated in ALS pathology, as studies have shown activation fragments of complement components C1q, C3 and C4 are increased in the serum, cerebrospinal fluid and neurological tissue (including spinal cord and motor cortex) of ALS patients [4].

In addition to evidence suggesting complement involvement in human ALS pathology, several studies have demonstrated the involvement of complement factors in animal models of ALS. Upregulation of the classical pathway complement components C1q and C4, as well as of the central factor C3, has been shown in human SOD1 transgenic rodent models of ALS [4]. Other studies have also shown upregulation of the major proinflammatory C5a receptor, CD88, during disease progression [10,11]. In addition, our group has shown that chronic administration of a specific CD88 antagonist in hSOD1^G93A^ transgenic rats delayed the onset of motor symptoms and increased survival compared with untreated animals [11]. Overall, these studies indicate that overactivation of the complement system and increased C5a-CD88 signalling contribute to the progression of disease in these animal models of ALS.

In the present study, we examined the expression and cellular location of major complement factors and regulators during defined disease stages in hSOD1^G93A^ mice in order to provide a comprehensive overview of complement’s involvement in ALS. Additionally, given the importance of C5a in disease pathology in ALS models [10,11], we also examined mRNA, protein levels, and the cellular localisation of C5, C5a and its cognate receptor, CD88, during disease progression. Our findings demonstrate a global dysregulation of complement, as disease progressed in these murine models of human ALS.

## Methods

### Ethical statement

All experimental procedures were approved by the University of Queensland Animal Ethics Committee (Permit Number: 227–09), and complied with the policies and regulations regarding animal experimentation and other ethical matters [12]. They were conducted in accordance with the Queensland Government Animal Research Act 2001, associated Animal Care and Protection Regulations (2002 and 2008), and the Australian Code of Practice for the Care and Use of Animals for Scientific Purposes, 7th Edition (National Health and Medical Research Council, 2004). ARRIVE guidelines have been followed in the preparation of the manuscript.

### Animals

Transgenic hSOD1^G93A^ mice (B6-Cg-Tg (SOD1-G93A) 1Gur/J) were obtained from Jackson Laboratory (Bar Harbor, ME, USA) and were bred on a C57BL/6J background to produce hSOD1^G93A^ mice and wild-type (WT) control mice. These hSOD1^G93A^ mice carry a high copy number of the mutated allele of the human (h) SOD1 gene. Female hSOD1^G93A^ and WT mice at four predefined stages of ALS were used in this study (Table 1). By the end stage of ALS, hSOD1^G93A^ mice display significant signs of hind-limb weakness, paralysis and loss of the righting reflex. All mice were anaesthetised with intraperitoneal injection of zolazapam (50 mg/kg, Zoletil; Lyppard, Melbourne, VIC, Australia) and xylazine (10 mg/kg, Xylazil; Lyppard) prior to the collection of tissue samples.

**Table 1:** Different stages defined in amyotrophic lateral sclerosis

**Stage**	**Age**	**Phenotype**
Pre-symptomatic	30 days postnatal	No signs of motor deficit
Onset	70 days postnatal	Initial signs of motor deficit (grip strength)
Mid-symptomatic	130 days postnatal	Weakness in hind-limb and tremor when suspended by the tail
End	150 to 175 days postnatal	Full paralysis of lower limbs and loss of righting reflex

### Weight measurements and behavioural tests

hSOD1^G93A^ and WT mice were weighed weekly at the same time of day (4:00 p.m.), from 42 days of age until end-stage when they lose their righting reflex. Two neuromotor tests, the Rota-rod and hind-limb grip strength test, were conducted on hSOD1^G93A^ and age-matched WT mice (detailed below). These tests were performed blinded to genotype.

#### *Rota-rod test*

Mice were tested for their motor coordination from 42 days of age using Rota-rod apparatus (Ugo Basile, Comerio, VA,Italy) at a constant speed of 20 rpm. Each mouse was given three attempts and the longest latency to fall was recorded; 180 seconds was chosen as the arbitrary cutoff time. One week prior to the test, mice were trained twice to remain on the Rota-rod apparatus to exclude differences in motivation and motor learning. In the training phase, mice were placed on the Rota-rod at a constant speed of 20 rpm for a maximum duration of 240 seconds [13].

#### *Hind-limb grip strength test*

A digital force gauge (Ugo Basile) was used to measure maximal muscle grip strength. The mice were held by their tail and lowered until the mice grasped the T-bar connected to the digital force gauge with their hind limbs. The tail is lowered until the body is horizontal and the mice are pulled away from the T-bar with a smooth steady pull until both of their hind limbs released the bar. The strength of the grip was measured in gram force. Each mouse was given 10 attempts and the maximum grip strength was recorded.

### Immunohistochemistry

hSOD1^G93A^ and WT mice were fixed by transcardiac perfusion with 2% sodium nitrite buffer (Ajax Finechem Pty Ltd, Cheltenham, VIC, Australia) followed by 4% paraformaldehyde (Sigma, St. Louis, MO, USA) in 0.1 M phosphate buffer pH 7.4. The lumbar spinal cords were collected and then placed in 4% paraformaldehyde for another 2 hours at 4°C. The lumbar spinal cords were embedded in optimal cutting temperature compound (Sakura Finetek, Torrance, CA, USA,) and snap frozen in liquid nitrogen. Serial transverse cryosections (16 μm) were collected on Superfrostplus slides (Menzel-Glaser, Braunschweig, Germany) for estimation of motor neuron numbers and fluorescence immunohistochemistry.

For the motor neuron numbers in the spinal cord, the sections are stained using 0.1% thionin (v/v) in acetic acid buffer solution (Sigma) for 3 minutes and processed as per our previous studies [14]. The lumbar lateral motor column extending from the second lumbar dorsal root ganglia to the sixth lumbar dorsal root ganglia was selected from our serial spinal sections, with the aid of the mouse spinal cord atlas [15]. Alpha-motor neurons within the lumbar lateral motor column were identified and counted on both sides of the spinal cord in every 10th section following previous guidelines [16-18]. The mouse genotypes were not made available to the researcher (JDL) conducting the counts until it was completed.

Fluorescence double-labelling was performed to localise the expression of C1q, C3b, C5 and its receptor CD88 with specific cell-type markers for motor neurons, astrocytes and microglia. All sections were rehydrated in PBS pH 7.4 and blocked in PBS containing 3% bovine serum albumin (BSA) or 3% donkey serum and 1% BSA for 1 hour at room temperature. Sections were then incubated overnight at 4°C with a combination of antibodies outlined in Table 2. All primary antibodies were diluted in PBS containing 1% BSA or 1% donkey serum. These sections were then washed 3× 10 minutes with PBS prior to incubation with an appropriate Alexa conjugated secondary cocktail: Alexa 555 goat anti-rat, Alexa 555 goat anti-mouse, Alexa 594 donkey anti-rat, Alexa 555 donkey anti-mouse, Alexa 488 goat anti-mouse, Alexa 488 goat anti-rabbit, Alexa 488 goat anti-rat and Alexa 488 donkey anti-goat (Invitrogen, Life Technologies, Mulgrave, VIC, Australia). All secondary antibodies were diluted in PBS containing 1% BSA or 1% donkey serum (1:1,000 for Alexa 555/594 and 1:600 for Alexa 488). Following 3× 5 minutes washes in PBS, the sections were incubated for 5 minutes in 4,6-diamidino-2-phenylindole (Invitrogen, Life Technologies). All sections were mounted with Prolong Gold Anti-Fade medium (Invitrogen, Life Technologies). IgG-negative controls (mouse IgG2a and rat IgG2a; AbD Serotec, Kidlington, UK) were used in place of primary antibodies to give a measure of nonspecific background staining. These IgG control antibodies were used at the same concentrations as and were of the same species as the primary antibodies listed above. Images were taken with a Zeiss LSM Meta 510 upright confocal microscope using a Plan-Apochromat 63× oil objective (Carl Zeiss Inc., Oberkochen, Germany).

**Table 2:** Summary of antibodies used for immunohistochemistry

**Antibody**	**Manufacturer**	**Dilution**	**In combination with**
Rat anti-mouse C1q	Hycult Biotechnology^1^	1:1,000	GFAP, Iba-1 and ChAT
Rat anti-mouse C3b	Hycult Biotechnology^1^	1:50	GFAP, Iba-1 and ChAT
Mouse anti-mouse C5	Hycult Biotechnology^1^	1:1,000	GFAP, CD11b, ChAT
Rat anti-mouse CD88	AbD Serotec^2^	1:250	GFAP, Iba-1, ChAT
Mouse anti-mouse GFAP	BD Biosciences^3^	1:1,000	C1q, C3b, C5, CD88
Rabbit anti-mouse Iba-1	Wako^4^	1:400	C1q, C3b and CD88
Rat anti-mouse CD11b	Abcam^5^	1:300	C5
Goat anti-mouse ChAT	Chemicon^6^	1:100	C1q, C3b, C5, CD88

*GFAP, glial fibrillary acidic protein; ChAT, choline acetyltransferase; Iba-1, ionized calcium-binding adapter molecule-1;*^1^*Hycult Biotechnology*, Netherlands**; **^2^ AbD Serotec; ^3^ BD Biosciences, Sydney, NSW, Australia; ^4^ Wako, Osaka, Japan; ^5^ Abcam, Sydney, NSW, Australia; ^6^ Chemicon, Billerica, MA, USA.

Quantification of immunofluorescence for C1q and C3b was performed on ~25 to 35 lumbar spinal cord sections (per animal; *n* = 3) spaced 160 μm apart and expressed as the percentage immunoreactive area per section. Staining procedures and image exposures were all standardised between genotype and between sections.

### Real-time quantitative PCR

Lumbar spinal cords from hSOD1^G93A^ and WT mice were collected into RNA*later* (Ambion, Life Technologies) and stored at -20°C for subsequent quantitative PCR analysis. Total RNA was isolated using an RNeasy Lipid Tissue extraction kit according to the manufacturer’s instructions (QIAGEN Inc, Alameda, CA, USA). After the total RNA was purified using Turbo DNAse treatment (Ambion, Life Technologies), cDNA was synthesised using the Stratagene RT kit (Agilent Technologies Inc, Santa Clara CA, USA). Commercially available gene-specific TaqMan probes (Applied Biosystems, Life Technologies) were used to amplify target gene of interest. All probes used are listed in Table 3. Relative target gene expression to glyceraldehyde-3-phosphate dehydrogenase (GAPDH) was determined using the formula 2^–∆CT^, where ∆Ct = (Ct target gene – Ct GAPDH) [19]. Final measures are presented as relative levels of gene expression in hSOD1^G93A^ mice compared with expression in WT controls.

**Table 3:** Taqman probes used for quantitative PCR

**Gene of interest**	**Catalogue number**
C1qB	Mm01179619_m1
C4	Mm00437896_g1
Factor B	Mm00433909_m1
C3	Mm01232779_m1
CD55	Mm00438377_m1
CD59a	Mm00483149_m1
C5	Mm00439275_m1
CD88	Mm00500292_s1
Glyceraldehyde-3-phosphate dehydrogenase	Mm99999915_g1
Mannose binding lectin 1	Mm00495413_m1
Mannose binding lectin 2	Mm00487623_m1

### Western blot analysis

Lumbar spinal homogenates from hSOD1^G93A^ and WT mice were resolved on a 10% SDS-PAGE gel and electrotransferred onto nitrocellulose membranes. Membranes were blocked in 2.5% skim-milk–Tris-buffered solution–0.1% Tween 20 for CD88 and 5% BSA–Tris buffered solution–0.1% Tween 20 for CD55 and were incubated overnight with one of the following antibodies; anti-CD88 (1:1,000; BMA Biomedical, Augst, Switzerland), or anti-CD55 (1:1,000; Hycult Biotechnology). All primary antibodies were diluted in 5% BSA–Tris buffered solution–0.1% Tween 20. Anti-CD88 was detected with goat anti-chicken horseradish peroxidase (HRP) (1:15,000; GE Healthcare, Pittsburgh, PA, USA) and anti-CD55 was detected with goat anti-rat HRP (1:10,000; GE Healthcare). These secondary antibodies were detected by enhanced chemiluminescence (ECL; Amersham, Pittsburgh, PA, USA). Blots were stripped and re-probed with anti-GAPDH, (1:15,000; Millipore, Billerica, MA, USA) and detected with sheep anti-mouse HRP (1:4,000; GE Healthcare) to ensure equal protein loading. Densitometric analyses of immunoreactive bands were performed by deducting background pixels from the grey-scale pixel density of the band multiplied by the area value using Image J software [20]. The integrated pixel value for each band was normalised to its corresponding anti-GAPDH band. The normalised integrated pixel values of hSOD1^G93A^ bands were compared with WT bands.

### *In situ* hybridisation

Synthesis of digoxigenin-labelled probes was performed using digoxigenin RNA labelling mix according to the manufacturer’s instructions (Roche, Brisbane, QLD, Australia) using PCR-amplified cDNA templates generated with primers specific for CD88: forward, TAATACGACTCACTATAGGGATCATCTACTCGGTGGTGTTCC and reverse, AATTAACCCTCACTAAAGGGGAGAGACCTTAGGAGTCGTCCA. Lumbar spinal cords from hSOD1^G93A^ and WT mice were collected and fixed overnight in 4% paraformaldehyde at 4°C. Samples were processed and embedded in optimal cutting temperature compound (Sakura Finetek), sectioned at 16 μm and probed with CD88 riboprobes and sense control as previously described [21].

### Enzyme-linked immunosorbent assay

Ninety-six-well plates (Greiner Bio-One, Frickenhausen, Germany) were precoated with monoclonal rat anti-mouse C5a capture antibody (Clone I52 – 1486; BD Pharmingen, San Diego, CA, USA) diluted in coating buffer (100 μM, NaHCO_3_, 34 μM Na_2_CO_3_, pH 9.5) overnight at 4°C in a sealed humidified container. This capture antibody is specific for a neo-epitope exposed only in mouse C5a/C5a desArg and does not cross-react with C5 [22,23]. Following the plate being blocked for 1 hour at room temperature with assay diluent (10% FCS/PBS), C5a standard and lumbar spinal cord homogenates was incubated for 2 hours at room temperature. The plates were subsequently incubated with biotinylated rat anti-mouse C5a detection antibody (clone I52-278; BD Pharmingen) for 1 hour at room temperature, and then incubated with streptavidin–HRP conjugate for 30 minutes at room temperature. Tetramethylbenzidine (Sigma) substrate was used as the chromogen and the plate was read at 450 nm. Levels of C5a in lumbar spinal cord samples were adjusted to micrograms per protein and expressed as nanograms of C5a per microgram of protein.

### Statistical analysis

All measures were performed using GraphPad Prism 5.0 (GraphPad Software Inc., San Diego, CA, USA). The statistical differences between hSOD1^G93A^ and WT mice for body weight, Rota-rod test and hind-limb grip strength test were analysed using a two-tailed Student’s *t* test at each time point. For the results from motor neuron counts, quantitative real-time PCR, western blotting, ELISA, statistical differences between hSOD1^G93A^ and WT mice were analysed using a two-tailed *t* test at each stage of disease progression. All data are presented as mean ± standard error of the mean and differences were considered significant when *P* ≤0.05.

## Results

### Motor deficits in hSOD1^G93A^ mice correlate with lumbar motor neuron loss during disease progression

To monitor the decline in neuromotor performance and loss of motor neurons during ALS progression in hSOD1^G93A^ mice, body weights, motor behavioural tests and motor neuron counts were performed. The onset of disease was defined as a stage in which a neuromotor deficit was measurable. In this study, hSOD1^G93A^ mice showed a decrease in their body weight, hind-limb grip strength and Rota-rod performance when compared with WT mice. The weight of the hSOD1^G93A^ mice reached the maximum at 133 days of age and was significantly decreased when compared with WT mice at 140 days (mean body weight, hSOD1^G93A^ = 20.4 ± 0.23 g and WT = 22.3 ± 0.25 g, *n* = 9, *P < 0.05, #*P* < 0.001; arrow in Additional file 1: Figure S1A).

The Rota-rod is a widely used measure of neuromotor performance in hSOD1^G93A^ mice [24]. In our study, both hSOD1^G93A^ and WT mice remained on the Rota-rod for the full duration of the test until 119 days when hSOD1^G93A^ mice showed ~30% reduction in the time remained on the Rota-rod (*n* = 12, *P < 0.05, #*P* < 0.001; arrow in Additional file 1: Figure S1B). Next, we measured maximal hind-limb grip strength as an alternate measure of neuromotor function [25,26]. At 70 days, hSOD1^G93A^ mice showed a significant reduction in grip strength (~35% reduction; *n* = 12, *P <0.05, +*P* <0.01, #*P* <0.001) when compared with WT mice (arrow in Additional file 1: Figure S1C). Our results suggest that the hind-limb grip strength test is a more sensitive measure of detecting motor deficit symptoms in hSOD1^G93A^ mice compared with weight loss and Rota-rod performance [26]. The Rota-rod test mainly evaluates balance and coordination, and does not necessarily reflect muscle denervation (that is, loss of muscle function [27]). By contrast, the decline in grip strength in the hSOD1^G93A^ mice closely correlated with the onset of lumbar motor neuron loss at 70 days (*n* = 6, ***P <0.001; in Additional file 1: Figure S1D). Post 70 days, we observed further declines in hind-limb grip strength presumably resulting from a progressive drop in lumbar motor neuron numbers up to end stage (in Additional file 1: Figure S1C, D).

### Components of the classical and alternate pathways of complement are upregulated along with decreased expression levels of complement regulators in hSOD1^G93A^ mice

Previous studies have identified various members of the complement system that are upregulated in ALS and in ALS animal models, but it is unclear which of the major complement pathways are being activated. To investigate this further, we examined the mRNA and protein levels for some of the key initiators of the complement pathways, the classical, alternate and lectin pathways, as well as the major complement regulators CD55 and CD59a [28].

**Figure 1:**
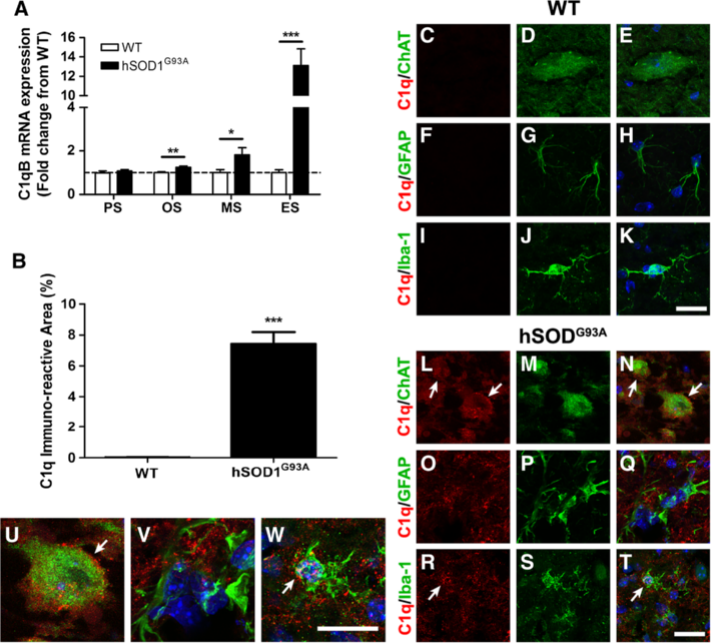
**Expression and localisation of C1q in hSOD1**^**G93A **^**and wild-type mice during disease progression. (A)** mRNA expression profile of C1qB in lumbar spinal cord of hSOD1^G93A^ mice relative to wild-type (WT) mice. Dashed line, baseline expression in WT controls at each time point. **(B)** Degree of immunolabelling for C1q significantly increased in the lumbar spinal cord of hSOD1^G93A^ mice at end stage when compared with WT mice. **(A**, **B)** Data expressed as mean ± standard error of the mean (*n* = 6 mice/group; *P <0.05, **P <0.01, ***P <0.001, Student *t* test). **(C)** to **(T)** Double immunolabelling of C1q (red) with cellular markers (green) for motor neurons (ChAT; **(C)** to **(E)** WT mice, **(L)** to **(N)** hSOD1^G93A^ mice), astrocyte (g*lial fibrillary acidic protein* (GFAP); **(F)** to **(H)** WT mice, **(O)** to **(Q)** hSOD1^G93A^ mice), and microglia (Iba-1; **(I)** to **(K)** WT mice, **(R)** to **(T)** hSOD1^G93A^ mice) in the ventral lumbar spinal cord of WT and hSOD1^G93A^ mice (end stage). There was minimal expression of C1q in WT **(C**, **F** and **I)** with marked increase in hSOD1^G93A^ mice **(L**, **O** and **R)**. In hSOD1^G93A^ mice, C1q was co-localised with ChAT-positive motor neurons **(**white arrows in **(L)** and **(N)** (detailed in **U**)**)**. There was little to no co-localisation of C1q with GFAP-positive astrocytes **(Q** (detailed in **V**)**)**, and minimal co-localisation with Iba-1-labelled microglia **(**white arrows in **R** and **T** (detailed in **W**)**)**. PS, pre-symptomatic; OS, onset; MS, mid-symptomatic; ES, end-stage. Scale bar for all panels = 20 μm.

**Figure 2:**
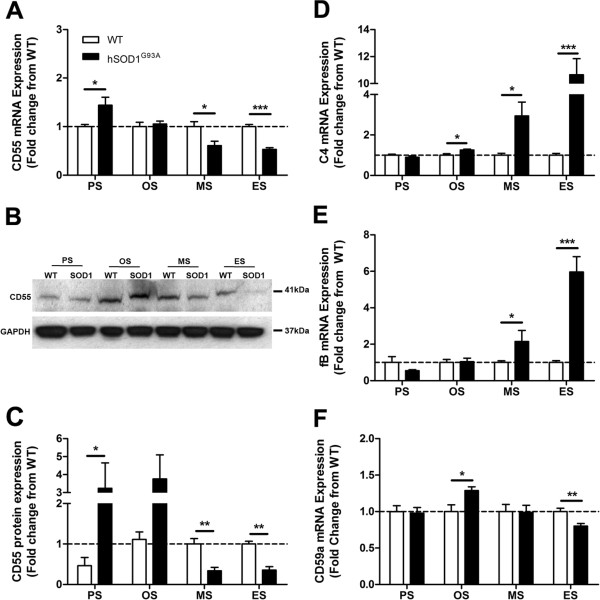
**Altered expression of complement components in hSOD1**^**G93A **^**and wild-type mice at different ages of disease progression. (A)** mRNA expression of CD55 in the lumbar spinal cord of hSOD1^G93A^ transgenic mice relative to age-matched wild-type (WT) mice at four different ages. **(B)** Representative western blot of CD55 with glyceraldehyde-3-phosphate dehydrogenase (GAPDH) in the lumbar spinal cord of hSOD1^G93A^ mice (SOD1) relative to age-matched WT mice, at different ages of disease progression. **(C)** Protein expression of CD55 determined by semi-quantitative densitometry in the lumbar spinal cord of hSOD1^G93A^ mice relative to age-matched WT mice at four different ages. **(D)** to **(F)** mRNA expressions of C4 **(**classical pathway, **D)**, factor B **(**alternative pathway, **E)** and CD59a **(**regulator, **F)** in lumbar spinal cord of hSOD1^G93A^ mice relative to age-matched WT mice at four different ages. **(A**, **C**, **D**, **E**, **F)** Dashed lines, baseline expressions in WT controls at each time point. Data expressed as mean ± standard error of the mean (*n* = 6 mice/group; *P <0.05, **P <0.01, ***P <0.001, Student *t* test).

The expression levels of initiating components of the classical pathway (C1qB and C4), the alternative pathway (factor B), the lectin pathway (mannose binding lectin 1 and 2), the central component common to all pathways, C3, and the complement regulators CD55 and CD59a were measured in the lumbar spinal cord of hSOD1^G93A^ mice during disease progression of ALS (30 to 175 days). This was achieved using one of or a combination of the following: quantitative real-time PCR, immunofluorescence and/or western blotting. C1qB and C4 transcripts were significantly increased by 1.2-fold and 1.3-fold at onset, 1.7-fold and 2.9-fold at mid-symptomatic disease, and 13.1-fold and 10.7-fold by end stage of disease in hSOD1^G93A^ mice when compared with WT mice respectively (*n* = 6, *P <0.05, **P <0.01 and ***P <0.001; Figures 1A and 2D). Upregulation of C1q at end stage was confirmed using immunofluorescence, where there was marked increase in hSOD1^G93A^ mice compared with WT mice (Figure 1B). We also observed that the marked increase of C1q in hSOD1^G93A^ mice was localised to motor neurons and activated microglia (white arrows in Figure 1L, N (detailed in 1U), and white arrows in Figure 1R, T (detailed in 1W)), compared with WT where little to no C1q was observed (Figure 1C, D, E and Figure 1I, J, K). We did not observe C1q on g*lial fibrillary acidic protein* (GFAP) expressing astrocytes in either hSOD1^G93A^ or WT mice (Figure 1O, P, Q (detailed in 1V) for hSOD1^G93A^ mice; Figure 1F, G, H for WT mice). Factor B showed a similar activation profile to that of C1qB and C4; namely, there was a 2.2-fold increase in mRNA at mid-symptomatic disease, and by end stage of disease, there was a 6.0-fold increase in hSOD1^G93A^ mice respectively compared with WT mice (*n* = 6, *P <0.05, ***P <0.001; Figure 2E).

**Figure 3:**
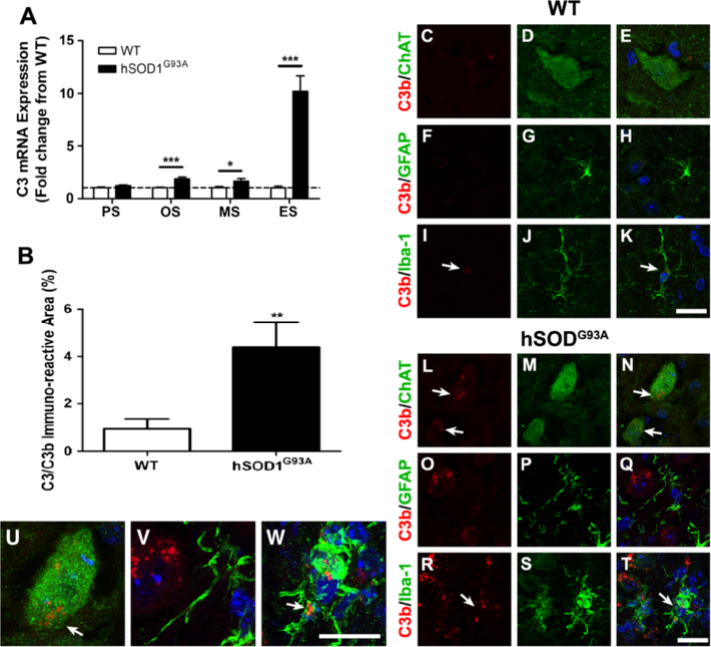
**Localisation and expression of C3/C3b in hSOD1**^**G93A **^**and wild-type mice during disease progression. (A)** mRNA expression profile of C3 in lumbar spinal cord of hSOD1^G93A^ mice relative to wild-type (WT) mice. Dashed line, baseline expression in WT controls at each time point. **(B)** Degree of immunolabelling for C3b significantly increased in the lumbar spinal cord of hSOD1^G93A^ mice at end stage when compared with WT mice. **(A**, **B)** Data expressed as mean ± standard error of the mean (*n* = 6 mice/group; *P <0.05, **P <0.01, ***P <0.001, Student *t* test). **(C)** to **(T)** Double immunolabelling of C3b (red) with cellular markers (green) for motor neurons (ChAT; **(C)** to **(E)** WT mice, **(L)** to **(N)** hSOD1^G93A^ mice), astrocyte (g*lial fibrillary acidic protein* (GFAP); **(F)** to **(H)** WT mice, **(O)** to **(Q)** for hSOD1^G93A^ mice), and microglia (Iba-1; **(I)** to **(K)** WT mice, **(R)** to **(T)** hSOD1^G93A^ mice) in the ventral lumbar spinal cord of WT and hSOD1^G93A^ mice (end stage). C3b immunolabelling was absent on motor neurons in WT mice **(C** to **E)**, but was present on motor neurons in hSOD1^G93A^ mice **(**white arrows in L and N (detailed in **U**)**)**. There was minimal co-localisation of C3b with Iba-1-labelled microglia in WT (white arrows in I and K). In hSOD1^G93A^ mice immunolabelling of C3b was evident in Iba-1-labelled microglia **(**white arrows, **R** and **T** (detailed in **W**)**)**. There was no co-localisation with C3b and GFAP-positive astrocytes in WT and hSOD1^G93A^ mice **(F** to **H** for WT, **O** to **Q** (detailed on V) for hSOD1^G93A^ mice**)**. PS, pre-symptomatic; OS, onset; MS, mid-symptomatic and ES, end-stage. Scale bars for all panels = 20 μM.

The central component of complement system C3 was also increased in hSOD1^G93A^ mice, but its expression profile only dramatically increased by end stage in hSOD1^G93A^ mice when compared with WT. Specifically, we observed 1.8-fold and 1.6-fold increases at onset and mid-symptomatic disease, with a dramatic 10.2-fold increase in C3 mRNA by end stage of disease when compared with WT mice (*n* = 6, *P <0.05 and ***P <0.001; Figure 3A). We then examined the expression and localisation of its activation fragment C3b in hSOD1^G93A^ and WT mice at end stage. Increased immunolabelling for C3b was observed in the lumbar spinal cords of hSOD1^G93A^ mice compared with WT mice at end stage (Figure 3B). In hSOD1^G93A^ mice, C3b deposition appeared primarily on motor neurons and microglia (white arrows in Figure 3L, N (detailed in Figure 3U), and white arrows in Figure 3R, T (detailed in Figure 3W) compared with WT, where there was little to no C3b staining (Figure 3C, D, E, and white arrows in Figure 3I, K). We did not observe C3b staining on GFAP expressing astrocytes in either hSOD^G39A^ or WT mice (Figure 3O, P, Q (detailed in Figure 3V) for hSOD1^G93A^ mice and Figure 3F, G, H for WT mice). Changes in mRNA expression level of mannose binding lectin 1 and mannose binding lectin 2, which are the initiating components of the lectin pathway, were not detectable in either hSOD1^G93A^ or WT mice (data not shown), suggesting this pathway plays a minor role in this model.

The regulators of complement system CD55 and CD59a were also investigated, because they are important in maintaining homeostasis and keeping the complement system in its proper physiological state. Specifically, CD55 and CD59a negatively regulate complement activation by accelerating C3 convertase decay and inhibiting the assembly of membrane attack complex respectively [29,30]. CD55 mRNA expression was initially increased by 1.4-fold at pre-symptomatic, and decreased at mid-symptomatic and end stage of disease by 0.4-fold and 0.5-fold respectively when compared with WT (*n*=6, *P <0.05 and ***P <0.001; Figure 2A). This was confirmed at protein level using western blotting, where a 41 kDa CD55 immunoreactive band was observed in all stages of hSOD1^G93A^ mice and their respective WT mice (Figure 2B, upper panel). Semi-quantitative densitometry analyses of these bands with respect to GAPDH loading controls (Figure 2B, lower panel), revealed increased CD55 protein in the lumbar spinal cord of hSOD1^G93A^ mice by 3.2-fold at pre-symptomatic and decreased by 0.7-fold at mid-symptomatic and 0.6-fold at end stage respectively when compared with WT mice (*n* = 4, *P <0.05 and ***P <0.001; Figure 2C). CD59a mRNA was also increased initially at onset by 1.3-fold and decreased at end stage of disease by 0.2-fold when compared with WT mice (*n* = 6, *P <0.05 and **P <0.01; Figure 2F).

Collectively, the above results suggest that regulation of complement system is perturbed, which leads to activation of classical and alternate pathways of complement system in the lumbar spinal cord of hSOD1^G93A^ mice, which may contribute to the disease progression of ALS.

### C5 is expressed by motor neurons but is not altered in hSOD1^G93A^ mice

**Figure 4:**
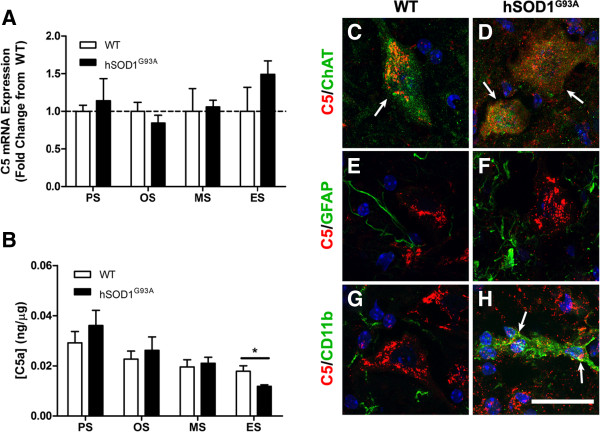
**Expression and localisation of C5 and C5a in hSOD1**^**G93A **^**and wild-type mice during disease progression. (A)** mRNA expression profile of C5 in lumbar spinal cord of hSOD1^G93A^ mice relative to wild-type (WT) mice. Dashed line, baseline expression in WT controls at each time point. **(B)** Protein expression of C5a in the lumbar spinal cord of hSOD1^G93A^ mice has decreased by end stage (ES) when compared with WT mice. **(A**, **B)** Data expressed as mean ± standard error of the mean (*n* = 6 mice/group; *P <0.05, Student *t* test). **(C)** to **(H)** Double immunolabelling for C5 (red) with cellular makers (green) for motor neurons (ChAT; **C** and **D**, arrows), astrocytes (g*lial fibrillary acidic protein* (GFAP), **E** and **F**), and microglia (CD11b; **G** and **H**), in the ventral lumbar spinal cord region of hSOD1^G93A^ mice **(D**, **F** and **H)** and WT mice **(C**, **E** and **G)** at end stage. Co-localisation of C5 with these cellular markers is seen as a merge of green and red **(**for example, white arrows in **C**, **D**, and **H)**. PS, pre-symptomatic; OS, onset; MS, mid-symptomatic; ES, end-stage. Scale bar for C to H = 20 μm.

C5a, the ligand for CD88, is rapidly generated from its precursor protein C5 following complement activation [31]. We therefore examined the mRNA expression of C5 and protein levels of C5a in hSOD1^G93A^ and WT mice by quantitative real-time PCR and ELISA respectively. C5 mRNA expression levels did not change in hSOD^G93A^ mice when compared with WT mice over the four ages examined (Figure 4A). Intriguingly, when we examined the protein expression levels of C5a we noted a steady decline in C5a levels with increasing postnatal age in both hSOD1^G93A^ and WT mice; however, by disease end stage the levels of C5a were significantly lower in hSOD1^G93A^ mice when compared with WT mice (*n* = 6, *P <0.05; Figure 4B).

Next, we immunostained lumbar spinal cords from hSOD1^G93A^ and WT mice for C5 with specific cellular markers for motor neurons (anti-ChAT), astrocytes (anti-GFAP), and microglia (anti-CD11b). C5 was clearly present in ChAT-positive lumbar motor neurons from end-stage hSOD1^G93A^ and WT mice (white arrows in Figure 4C, D), but not in GFAP-positive astrocytes (Figure 4E, F). For microglia, we did not see any C5 in CD11b-positive microglia in WT spinal cords (Figure 4G), but we did see some activated microglia (enlarged cell shape with thickening of proximal processes and decrease in ramification of distal branches [32]) expressing low amounts of C5 in the spinal cords from end-stage hSOD1^G93A^ mice (white arrows; Figure 4H).

### CD88 is upregulated during disease progression in hSOD1^G93A^ mice

Previous studies have shown an increase in CD88 expression in multiple rodent models of ALS [10,11]; hence this study aimed to investigate whether there were similar differences in expression of CD88 between hSOD1^G93A^ and WT mice during disease progression of ALS.

**Figure 5:**
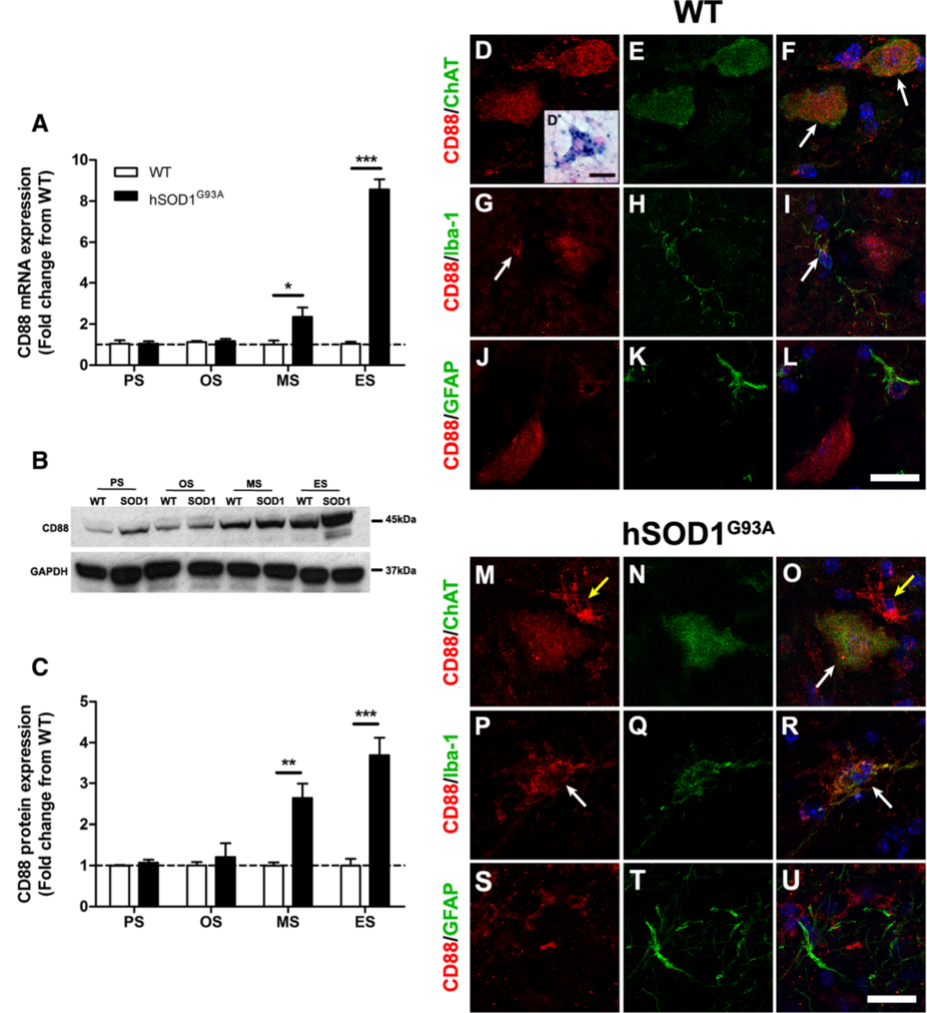
**Expression and localisation of CD88 in hSOD1**^**G93A **^**and wild-type mice at four different ages. (A)** mRNA expression of CD88 in the lumbar spinal cord of hSOD1^G93A^ mice relative to age-matched wild-type (WT) mice at four different ages. **(B)** Representative western blot of CD88 with glyceraldehyde-3-phosphate dehydrogenase (GAPDH) in the lumbar spinal cord of hSOD1^G93A^ (SOD1) mice relative to age-matched WT mice at different ages. **(C)** Protein expression of CD88 determined by semi-quantitative densitometry in the lumbar spinal cord of hSOD1^G93A^ mice relative to age-matched WT mice at four different ages. **(A)**, **(C)** Dashed lines, baseline expression in WT controls at each time point; data expressed as mean ± standard error of the mean (*n* = 6 mice/group; *P <0.05, **P <0.01, ***P <0.001, Student *t* test). **(D)** to **(U)** Double immunolabelling of CD88 (red) with cellular markers (green) for motor neurons (ChAT; **(D)** to **(F)** WT mice, **(M)** to **(O)** hSOD1^G93A^ mice), microglia (Iba-1; **(G)** to **(I)** WT mice, **(P)** to **(R)** hSOD1^G93A^ mice), and astrocytes (g*lial fibrillary acidic protein* (GFAP); **(J)** to **(L)** WT mice, **(S)** to **(U)** for hSOD1^G93A^ mice) in the ventral lumbar spinal cord of WT and hSOD1^G93A^ mice at end stage. CD88 was co-localised with ChAT-positive motor neurons **(F**, **O**, white arrows**)**. **(D′)** CD88 mRNA transcript within lumbar motor neurons (determine by large cell size and location within the ventral horn). In hSOD1^G93A^ mice, immunolabelling of CD88 also evident on other cell types, indicated by lack of co-localisation with anti-ChAT **(**yellow arrows in **M** and **O)**. **(G)**, **(I)** White arrows, small amount of CD88 staining within nonactivated microglia in WT mice, with increased CD88 expression on activated microglia in hSOD1^G93A^ mice **(P** and **R**, white arrows**)**. PS, pre-symptomatic; OS, onset; MS, mid-symptomatic; ES, end stage. Scale bars for all panels = 20 μM.

The mRNA expression levels for CD88 in the lumbar spinal cord of hSOD1^G93A^ mice were normalised and compared with WT mice during disease progression (30 to 175 days) using quantitative real-time PCR. CD88 expression was increased by 2.3-fold at mid-symptomatic, and by 8.6-fold at end stage of disease respectively compared with WT mice (*n* = 6, *P <0.05 and ***P <0.001; Figure 5A). The protein expression level of CD88 in the lumbar spinal cords of hSOD1^G93A^ and WT mice were measured using western blotting. A 45 kDa CD88 immunoreactive band was observed in all stages of hSOD1^G93A^ mice and their respective WT mice (Figure 5B, upper panel). Semi-quantitative densitometry analyses of these bands with respect to GAPDH loading controls (Figure 5B, lower panel) revealed increased CD88 protein in the lumbar spinal cord of hSOD1^G93A^ mice by 2.6-fold and 3.7-fold at mid-symptomatic and end stage respectively when compared with WT mice (*n* = 6, *P <0.05 and ***P <0.001; Figure 5C).

### CD88 is localised to motor neurons and activated microglia with minimal localisation to astrocytes in hSOD1^G93A^ mice

Next, we aimed to determine the cellular localisation of CD88 that has contributed to the increased expression seen in hSOD1^G93A^ mice. To achieve this, we performed immunolabelling for CD88 on lumbar spinal cord sections from hSOD1^G93A^ mice and WT mice. These sections were immunostained for CD88 and with specific cellular markers to identify motor neurons (anti-ChAT), microglia (anti-Iba-1) and astrocytes (anti-GFAP).

In WT mice, CD88 staining was observed on lumbar motor neurons (Figure 5D). CD88-stained cells were readily identified as motor neurons due to their large size, location and distinctive morphology [17]. This was confirmed by double-labelling with the motor neuron marker ChAT (white arrows in Figure 5F). CD88 immunostaining was localised predominantly to the motor neurons’ soma (Figure 5D, E, F). To further support these immunohistochemical findings, we confirmed that motor neurons expressed CD88 mRNA transcripts by *in-situ* hybridisation (Figure 5D′, inset). Next, we examined whether CD88 immunostaining was present on surrounding microglia and astrocytes in sections of WT lumbar spinal cords. We observed minimal co-localisation of CD88 to Iba-1-positive microglia (white arrows in Figure 5G,I), but none to GFAP-positive astrocytes (Figure 5J, K, L).

Following this demonstration of CD88 immunolabelling in WT lumbar spinal cord, we then investigated CD88 cellular localisation in hSOD1^G93A^ mice. CD88 was also expressed on the few remaining motor neurons seen at the end-stage of disease (white arrow in Figure 5O). By contrast to WT lumbar spinal cords, we observed prominent CD88 immunostaining on other cellular structures surrounding motor neurons in the lumbar spinal cords of hSOD1^G93A^ mice by the end stage of disease (for example, yellow arrows in Figure 5M, O).

This additional CD88 immunoreactivity was investigated in hSOD1^G93A^ end-stage mice, using microglia marker Iba-1 and astrocyte marker GFAP. By contrast to Iba-1-positive microglia in WT mice, where these cells expressed little observable CD88 and appeared to be nonactivated (that is, small size with slender processes; [32]; Figure 5H, I), Iba-1-positive microglia in hSOD1^G93A^ mice demonstrated an activated morphology with increased expression of CD88 (white arrows in Figure 5P, R).

As expected, astrocytes were seen to increase in numbers as disease progressed in hSOD1^G93A^ mice [33]. This was noted by increased GFAP immunolabelling in the lumbar spinal cord of hSOD1^G93A^ mice at end stage compared with WT mice (Figure 5K, L for WT, and Figure 5T, U for hSOD1^G93A^ mice). Minimal CD88 co-localisation to GFAP-positive astrocytes was observed in WT mice (Figure 5J, K, L) and at the end stage of disease in hSOD1^G93A^ mice (Figure 5S, T, U).

## Discussion

While the pathogenesis of ALS is still unclear, there is persuasive evidence that complement factors are involved in promoting disease progression. Previous studies have demonstrated that C1q and C3 mRNA expression are significantly increased during ALS progression in hSOD1^G93A^ mice [4]. In addition, upregulation of CD88 has also been observed in numerous neurodegenerative diseases in rodents [34-37], including ALS [10,11], so it is plausible to propose that the complement system could be involved in the pathophysiology of ALS. The present study demonstrates that components of the classical and alternative complement pathways are upregulated during the course of disease progression in hSOD1^G93A^ mice, and that C5a receptor CD88 expression level is also increased. In addition, we found a reduction in two major regulatory inhibitors of complement activation as the disease worsened, which is suggestive of a progressive dysregulation of complement in this model. Furthermore, we show that C5, the precursor of C5a, is expressed predominantly by motor neurons, suggesting that diseased motor neurons could be a major source of C5a generation during disease progression. This local complement self-signalling in the central nervous system might therefore contribute to motor neuron death in hSOD1^G93A^ mice as shown previously for cortical neurons [22]. Taken together, our results indicate that motor neurons may generate C5a under stress, and that this may promote self-damage under disease conditions that exist in ALS.

### Classical and alternate complement pathways are activated in ALS progression in hSOD1^G93A^ mice

The present study provided evidence for the activation of classical (C1qB and C4) and alternate (factor B) pathways of the complement system in the lumbar spinal cord of hSOD1^G93A^ mice during ALS disease progression. This is consistent with numerous studies in mouse models of ALS and human patients where increased levels of C1q, C3 and C4 have been found [4,38]. Furthermore this study also extended upregulation of C1qB and C3 mRNA expression in previous studies to protein levels and localisation where C1q and C3b immunolabelling was increased in hSOD1^G93A^ mice and expressed on motor neurons and microglia compared with WT. This may suggest that upregulation of these components could assist in the removal of dying motor neurons via opsonisation, during disease progression in hSOD1^G93A^ mice [39].

It is also possible that cell fragments or protein aggregates from dying motor neurons could lead to complement activation in the degenerating spinal cord [28]. In our study, complement activation was seen at disease onset (P70) and was restricted to the areas of motor neuron death in the spinal cord of hSOD1^G93A^ mice. Other studies have demonstrated that complement components C1q and C3b are located at the neuromuscular junction during the early stages of disease (P47) in hSOD1^G93A^ mice [40]. These findings are consistent with the idea that C1q and C3b may contribute to the cellular destruction of motor nerve terminals in these mice [41-43]. Taken together, these findings and our own are consistent with the hypothesis that the early loss of motor neuron terminals is followed by the subsequent death of motor neurons within the spinal cord. However, there is current debate about the initiating site of degeneration in the cortical-motor system (upper motor neurons [44]) versus peripheral (neuromuscular junction [41]). Future studies could contrast complement activation temporally at these different sites to determine the initiating site of complement-mediated neurodegeneration.

We also showed decreased mRNA expression levels of complement regulators CD55 and CD59a at later stages of the disease, which suggests that the homeostasis of the complement system is perturbed, which may lead to dysregulation and overactivation of the complement system. This supports other studies, which have shown that deficiency in CD55 and CD59a exacerbates neuronal degeneration [45-47]. Our findings are also consistent with those of Heurich and colleagues [40], where decreased (but nonsignificant) levels of CD55 mRNA were observed during the later stages of disease in hSOD1^G93A^ mice. In the present study, we also confirmed the mRNA changes in CD55 at the protein level, which similarly showed decreased CD55 levels at later stages of disease. Interestingly, we also observed an initial increase in the mRNA expression levels of CD55 and CD59a during early stages of disease. This may indicate an early negative feedback mechanism to delay the activation of complement in host cells, but this needs further investigation.

Collectively, our data add further support to the notion that complement activation may play an important role in accelerating motor neuron loss and ultimately in progression of ALS.

### C5 is expressed by wild-type and hSOD1^G93A^ motor neurons during disease progression

C5, the precursor of C5a, is expressed by motor neurons in both WT and hSOD1^G93A^ mice. This suggests that motor neurons are a major source of C5 generation in this tissue. We recently showed murine cortical neurons also expressed endogenous C5, and generated C5a in response to ischaemia, which contributed to neuronal cell death [22]. It is plausible the same phenomenon is occurring in the hSOD1^G93A^ mouse, where stressed and dying motor neurons generate their own C5a, to act in an autocrine fashion by binding to CD88, present on these neurons, to promote their death. Indeed, C5a has been suggested to directly cause neuronal cell death in a separate model of ALS [10]. C5a protein level in the lumbar spinal cord only appeared to be significantly decreased by end stage in hSOD1^G93A^ mice when compared with WT mice. This could be due to fewer motor neurons (a source of C5/C5a), and the increased CD88 receptor levels by surrounding activated microglia as disease progressed. Increased CD88 on these cells would act to internalise and degrade C5a post activation of its receptor [48], which could account for the decline in C5a levels over time. The consequence of C5a–CD88 signalling in WT motor neurons is not yet fully understood and will need further investigation.

### C5a receptor CD88 is upregulated during disease progression in hSOD1^G93A^ mice

The present study provided evidence for a pathophysiological role of CD88 in hSOD1^G93A^ mice. Specifically, a significant increase in CD88 protein was observed at mid-symptomatic and end stage in hSOD1^G93A^ mice. This is in agreement with our previous studies conducted in hSOD1^G93A^ rats [11]. The increase in CD88 protein in hSOD1^G93A^ mice also parallels observations in other models of neurodegenerative diseases, such as Huntington’s disease and Alzheimer’s disease [35,36]. Taken together, this suggests that complement activation is a generalised response to neuronal injury in neurodegenerative diseases. Furthermore, the ability of CD88 antagonists to attenuate both neurodegeneration and disease progression in rat models of ALS and Huntington’s disease, and in mouse models of Alzheimer’s disease, further suggests that increased CD88 activation actively contributes to neurodegeneration [11,37,49].

In addition to an increase in CD88 in hSOD1^G93A^ mice, the present study observed CD88 on motor neurons in WT mice, as well as in hSOD1^G93A^ mice. The fact that CD88 was found on WT motor neurons suggests that it may play a non-inflammatory role in motor neuron function. Indeed, studies including our own, have shown CD88 is also present on other neurons within the brains of WT adult mice, including pyramidal neurons in the CA subfields of the hippocampus and neocortex, and Purkinje cells in the cerebellum [50,51]; and CD88 has also been documented to be expressed on human motor neurons [10]. Hence the physiological significance of C5a receptor presence in motor neurons awaits further study.

Previous studies have shown that motor neuron death in animal models of ALS is exacerbated by toxic signals emanating from non-neuronal neighbouring cells (astrocytes and microglia), via an inflammatory response that accelerates disease progression [52,53]. The present study also showed upregulation of CD88 on activated microglia, but minimal expression on astrocytes in hSOD1^G93A^ mice. The latter is in contrast to our previous study conducted in hSOD1^G93A^ rats, where CD88 was expressed primarily on proliferating astrocytes [11], but is in support of other studies that show CD88 on microglia in other neurodegenerative diseases [36,54] The differential expression of CD88 on these proliferating glial cells between hSOD1^G93A^ rat and mouse models may suggest a differential role for CD88 in these two species.

The strong co-localisation of CD88 with activated microglia in hSOD1^G93A^ mice suggests that CD88-activated microglia contribute to the propagation of disease as opposed to the aetiology of the disease. This is supported by previous studies where transplanted WT microglia produced no delay of disease onset but survival was greatly extended through slowing of disease progression in hSOD1^G93A^ mice [55]. The exact mechanism by which C5a–CD88 signalling in microglia plays a role in neurodegeneration is still unknown, but may involve the release of reactive oxygen species through NADPH oxidase, or proinflammatory cytokines, which have been shown to be upregulated in ALS [56].

## Conclusions

In summary, the present study has demonstrated the upregulation of classical and alternative pathway complement components, together with decreased levels of complement regulators, suggesting that complement activation and/or its dysregulation may play an important role in motor neuron loss and ultimately in progression of ALS. Expression of C5a receptor CD88 was upregulated in hSOD1^G93A^ mice, and the increased expression of CD88 in end-stage hSOD1^G93A^ mice appears to be due to increased microglial CD88 expression. Taken together, these results indicate that CD88 may play an important role in the pathophysiology of ALS. These results pave the way for preliminary pharmacological experiments using specific downstream complement inhibitors in hSOD1^G93A^ mice, such as C5 inhibitors or antagonists to C5a receptor that could conceivably be extended to humans with positive therapeutic outcomes.

## Abbreviations

ALS: Amyotrophic lateral sclerosis; BSA: Bovine serum albumin; ELISA: Enzyme-linked immunosorbent assay; FCS: Foetal calf serum; GAPDH: Glyceraldehyde-3-phosphate dehydrogenase; GFAP: Glial fibrillary acidic protein; HRP: Horseradish peroxidase; PBS: Phosphate-buffered saline; PCR: Polymerase chain reaction; WT: Wild type.

## Competing interests

The authors declare that they have no competing interests.

## Authors’ contributions

PGN, SMT and TMW conceived the project. JDL performed most of the experiments. NAK and JNT performed additional experiments. JDA and BJT provided additional tissues. JDL, PGN and TMW wrote the paper. All authors read and made comment on the manuscript during its drafting. All authors read and approved the final manuscript.

## Supplementary Material

Additional file 1: Figure S1 Showing the decline in motor performance during ALS progression correlates with lumbar motor neuron loss in the lumbar spinal cord of hSOD1^G93A^ mice. **(A)** Significant weight loss of hSOD1^G93A^ mice when compared with wild-type (WT) control mice at 140 days of age (arrow, *n* = 12, *P <0.05, #*P* <0.001, Student *t* test). **(B)**, **(C)** Significant reduction in time spent on Rota-rod and hind-limb grip strength for hSOD1^G93A^ versus WT mice, at 119 days and 70 days respectively (arrows, *n* = 12, *P <0.05, +*P* <0.01, #*P* <0.001, Student *t* test). **(D)** Lumbar motor neuron loss in hSOD1^G93A^ mice when compared with WT control mice at 70 days of age onwards (*n* = 6, ***P <0.001, Student *t* test). The decline in motor neuron number at 70 days correlates with the onset of loss of hind limb muscle strength at this same age (**C**). Data expressed as mean ± standard error of the mean. PS, pre-symptomatic (30 days postnatal (P30)); OS, onset (70 days postnatal (P70)); MS, mid-symptomatic (130 days postnatal (P130)); ES, end-stage (175 days postnatal (P175)). Click here for file
