# Supplementary material for: Dysregulation of the complement cascade in the hSOD1G93A transgenic mouse model of amyotrophic lateral sclerosis
Source: J Neuroinflammation. 2013 Sep 26;10:119. doi: 10.1186/1742-2094-10-119 (PMC3850877; doi:10.1186/1742-2094-10-119)
Supplement: Additional file 1: Figure S1 — Showing the decline in motor performance during ALS progression correlates with lumbar motor neuron loss in the lumbar spinal cord of hSOD1G93A mice. (A) Significant weight loss of hSOD1G93A mice when compared with wild-type (WT) control mice at 140 days of age (arrow, n = 12, *P <0.05, #P <0.001, Student t test). (B), (C) Significant reduction in time spent on Rota-rod and hind-limb grip strength for hSOD1G93A versus WT mice, at 119 days and 70 days respectively (arrows, n = 12, *P <0.05, +P <0.01, #P <0.001, Student t test). (D) Lumbar motor neuron loss in hSOD1G93A mice when compared with WT control mice at 70 days of age onwards (n = 6, ***P <0.001, Student t test). The decline in motor neuron number at 70 days correlates with the onset of loss of hind limb muscle strength at this same age (C). Data expressed as mean ± standard error of the mean. PS, pre-symptomatic (30 days postnatal (P30)); OS, onset (70 days postnatal (P70)); MS, mid-symptomatic (130 days postnatal (P130)); ES, end-stage (175 days postnatal (P175)). [file 1742-2094-10-119-S1.pdf]

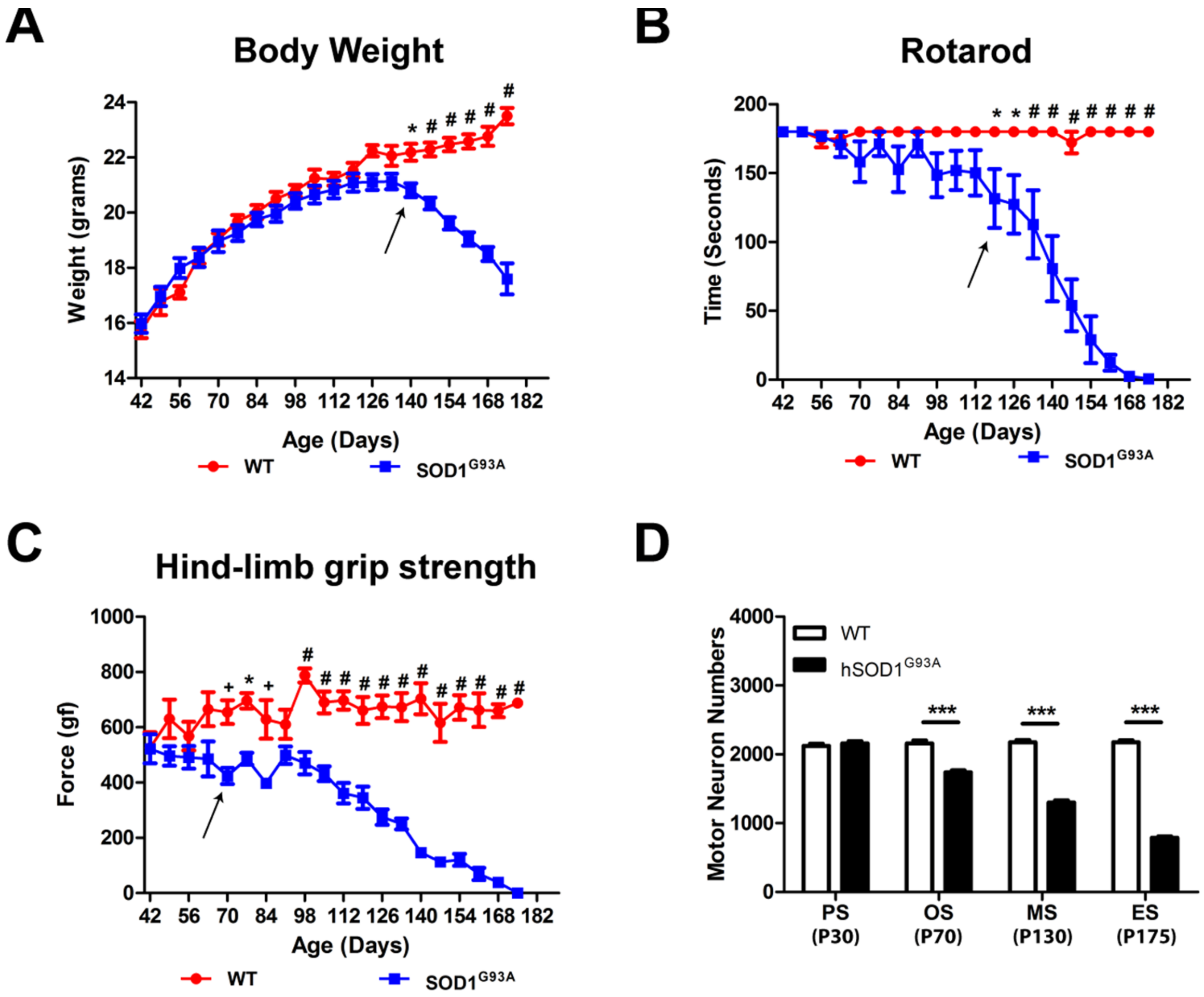


**Supplementary Figure 1. Decline in motor performance during ALS progression correlates with lumbar motor neuron loss in the lumbar spinal cord of hSOD1$^{G93A}$ mice.** **A** shows significant weight loss of hSOD1$^{G93A}$ mice when compared to wild type (WT) control mice at 140 days of age (arrow, $n = 12$, * $P < 0.05$, # $P < 0.001$, student $t$-test). **B** and **C** show significant reduction in time spent on rota-rod and hind-limb grip strength for hSOD1$^{G93A}$ versus WT mice, at 119 days and 70 days respectively (arrows, $n = 12$, * $P<0.05$, + $P<0.01$, # $P<0.001$, student $t$-test). **D** shows lumbar motor neuron loss in hSOD1$^{G93A}$ mice when compared to WT control mice at 70 days of age onwards ($n = 6$, *** $P<0.001$, student $t$-test). The decline in motor neuron number at 70 days correlates with the onset of loss of hind limb muscle strength at this same age (**C**). Data are expressed as mean ± SEM. PS = pre-symptomatic (30 days postnatal [P30]); OS = onset (70 days postnatal [P70]); MS = mid-symptomatic (130 days postnatal [P130]) and ES = end-stage (175 days postnatal [P175]).
